# Inflammatory bowel disease (IBD)-like disease in a case of a 33-year old man with glycogenosis 1b

**DOI:** 10.1186/s12876-015-0271-9

**Published:** 2015-04-08

**Authors:** Magdalena Sarah Volz, Mani Nassir, Christoph Treese, Moritz von Winterfeld, Ursula Plöckinger, Hans-Jörg Epple, Britta Siegmund

**Affiliations:** 1 Department of Medicine I (Gastroenterology, Rheumatology, Infectious Diseases), Charité – Universitätsmedizin Berlin, Berlin, Germany; 2 Institute of Pathology, Charité – Universitätsmedizin Berlin, Berlin, Germany; 3 Interdisciplinary Center of Metabolism: Endocrinology, Diabetes and Metabolism, Charité – Universitätsmedizin Berlin, Berlin, Germany

**Keywords:** Crohn’s disease, IBD-like disease, Stenosis, Glycogenosis, Glycogen storage disease, Granulocyte colony stimulating factor, G-CSF

## Abstract

**Background:**

Inflammatory bowel disease (IBD)-like conditions in glycogen storage disease (GSD) type Ib have been predominantly described in children. Signs and symptoms of GSD type Ib are hypoglycemia, pancytopenia and hepatosplenomegaly. Based on few published cases, there is evidence that granulocyte-colony stimulating factor (G-CSF) in patients with glycogenosis–related pancytopenia might ameliorate the IBD-like disease through leukocyte increase.

**Case presentation:**

Here we firstly describe a case of an adult 33-year-old Caucasian male patient with GSD type Ib accompanied with IBD-like disease with persistent pancytopenia despite moderate-dose G-CSF treatment. Recent vomiting and abdominal discomfort were due to a high-grade stenosis in the transverse colon. A dose increase of the G-CSF successfully normalized his leukocyte count. However, the stenosis worsened and surgical therapy was needed.

**Conclusion:**

We suggest that symptomatic patients with GSD type Ib should undergo endoscopic examination in order to detect IBD-like disease and to initiate early treatment.

## Background

Glycogen storage disease Ib (GSD Ib) is characterized by deficiency of glucose-6-phosphate translocase (Gene: SLC37A4) [1] inherited as an autosomal recessive trait. Clinically the disease is characterized by deficient glycogenolysis resulting in hypoglycemia, hypertriglyceridemia, phosphate deficiency and lactate acidosis and hepatosplenomegaly. Pancytopenia may occur with GSD Ib [2]. So far, inflammatory bowel disease (IBD)-like conditions has been predominantly described in children with GSD Ib with mean age of approximately 5.9 years [3-5]. This case reports of an adult 33-year-old man.

## Case presentation

**Figure 1:**
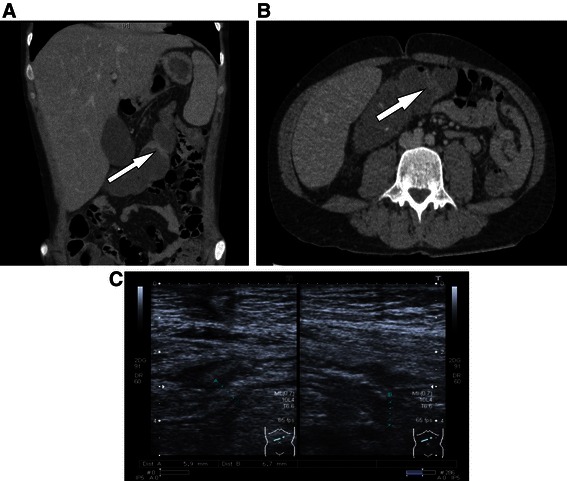
**Radiographic pictures of the stenosis. 1A** and **1B**: CT-scan of the stenosis of the transverse colon (**1A**: frontal view; **1B**: horizontal view). **1C**: Ultrasound picture of the stenosis. Stenosis was 5.9 mm × 6.7 mm in diameter. The intestinal wall appeared thickened and hypoechoic. There was no intra- or extra-mural vascularization present consistent with level I according to the Limberg classification.

**Figure 2:**
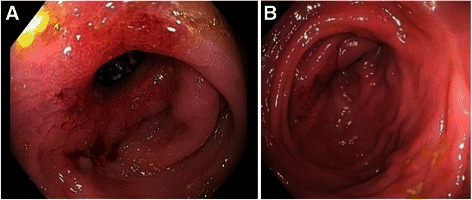
**Endoscopic pictures of the stenosis. 2A**: First colonoscopy with macroscopic inflammation. **2B**: Second colonoscopy with increased stenosis.

Here we report a 33-year-old Caucasian man with GSD Ib presented at our emergency room complaining of abdominal pain, nausea and vomiting. Onset of symptoms was acute. The patient reported not to know similar conditions before except diffuse pain due to hypoglycemia, which he described differently. The patient had an irregular heterozygous compound gene mutation (c.[1108_1109delCT(;)1189 + 1G > C]; protein level: p.[Leuc370Valfs*53(;)]; in exons 10), suggestive to be disease relevant. No other family member suffered from GSD. He reported regular defecation of mushy consistency without blood three times a day. Physical examination revealed in spite of pronounced hepatomegaly and cachexia, normal oral and anal mucosa as well as normal height and intelligence (occupation: sales representative in the IT industry). Despite regular cornstarch ingestion of 90 g/every 4 h, the glucose concentration at first presentation was 34 mg/dl, lactate concentration was 134 mg/dl (ULN = 20 mg/dl), and C-reactive protein was 13.5 mg/l (ULN = 5 mg/l). Even though the patient was on continuous granulocyte colony stimulating factor (G-CSF) therapy (48 million IU/every other day) leukopenia prevailed (leukocytes 0.93/nl, neutrophils 0.76/nl, hemoglobin 10.0 g/dl, thrombocytes 125/nl). Initially intravenous glucose infusion improved his abdominal symptoms. A diagnostic CT-scan to exclude ischemia demonstrated hepatosplenomegaly, a feature of GSD Ib, and a short thickening of the intestinal wall at the transverse colon (Figure 1A and B). Ultrasound examination of the abdomen confirmed the stenosis. At the location of the stenosis the entire intestinal wall was 6 mm in diameter. There was no intestinal wall vascularization, consistent with grade I of IBD according to the Limberg classification [6] (Figure 1C). A colonoscopy again revealed a short stenosis in the transverse colon which could not be passed with a 12.8 mm colonoscope (Figure 2A). The stenosis appeared scarred with some inflammatory changes. Histopathological examination of the biopsy showed hyperplastic and polypoid mucosa. Gastroscopy of the upper gastrointestinal tract was without pathologies. Due to the severe leukopenia/neutropenia, immunosuppressive medication, such as steroids or TNF-alpha antibodies, was not considered a treatment option. Instead, we increased the dosage of G-CSF to 30 million IU/d in order to reach a higher leukocyte count and thus, to control the mucosal inflammation. The decision was based on evidence for a beneficial effect of G-CSF in glycogenosis-associated IBD-like disease [3] as well as a previous randomized controlled trial in patients with Crohn’s disease showing that GM-CSF was able to decrease disease activity and to improve quality of life [7].

**Figure 3:**
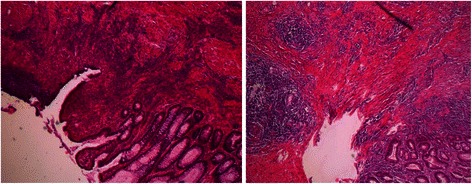
**Histological picture.** Pathological picture of instantaneous section showing chronic, florid ulcerous inflammation as well as a hypertrophy of the muscular layer and massive fibrosis consistent with a scarring stenosis.

After five weeks on high-dose G-CSF abdominal pain had receded, yet defecation was still mushy. Leucocytes had normalized (5-7/nl), while anemia and thrombocytopenia persisted (hemoglobin: 11.8 g/dl (LLN = 13.5 g/dl); thrombocytes: 84/nl (LLN = 150/nl)). C-reactive protein (0.7 mg/l) was normal as was calprotectin (<50 mg/kg). However, colonoscopy revealed a progressive, predominantly scarring stenosis with approximately 4 mm in diameter (Figure 2B). Thus, surgical therapy was decided upon. A laparoscopic segmental resection of the transverse colon with end-to-end-anastomosis was performed. The instantaneous section showed chronic, florid ulcerous inflammation, hypertrophic muscular layer and massive fibrosis consistent with a scarring stenosis. No signs of malignancy were observed (Figure 3). Surgery was complicated by insufficiency of the anastomosis resulting in an intra-abdominal abscess and subsequent surgery. Eighteen days after the second surgery the patient could be discharged from the hospital on his former G-CSF schedule.

## Discussion

We describe a case of an IBD-like disease in an adult patient with GSD type Ib. This condition is caused by a deficiency of the enzyme glucose-6-phosphate translocase as well as pancytopenia [1,2]. To the best of our knowledge, only few cases of IBD-like disease in GSD type Ib, mainly in children, have been described so far [5,8-14]. The patient in our case was 33-year-old before he presented symptoms of an IBD-like disease. This might be due to his continuous G-CSF treatment, since neutrophil count has been maintained higher and potentially delayed onset of IBD-like disease. On the other hand, his irregular heterozygous compound gene mutation could be an explanation why he got symptomatic as an adult and not as a child. Overall, IBD is thought to be associated with GSD type Ib, and leukopenia/neutropenia may play a role in the pathogenesis of intestinal inflammation [5]. This assumption is supported by evidence that colony stimulating factors can improve IBD-like disease in patients with GSD type Ib [3,7,15,16]. In a previous report, it was shown that G(M)-CSF not only increased neutrophil count, but also improved intestinal inflammation as indicated by radiological findings and clinical symptoms of two young patients with GSD-associated IBD-like disease [15]. The authors suggested that neutrophil deficiency might contribute to the development of this phenotype by facilitating acute and chronic infection in the gut mucosa leading to subsequent inflammation [15]. Alternatively, abnormal bowel metabolism resulting from translocase deficiency in the mucosal cells may be an explanation. In contrast to these previous reports, G-CSF did not improve the IBD-like disease in the present case of an adult patient suffering from GSD type Ib. Moreover, despite a normalization of leucocytes/neutrophils in response to the dose increase of G-CSF treatment, his abdominal stenosis worsened. This may be due to i) the fact that despite normalization of leukocyte counts, dysfunctional glucose-6-phosphatase deficient neutrophils are less effective in dealing with inflammatory processes [15], ii) the acute inflammatory stage of the IBD-like disease had already passed and a mainly scarring stenosis remained, when the patient presented as indicated by the scarred endoscopic and pathologic appearance.

## Conclusion

Therefore, we suggest that even in adult patients with GSD type Ib abdominal symptoms, normally interpreted as related to hypoglycemia or lactate acidosis, should include the differential diagnosis of an IBD-like disease. Immediate abdominal ultrasound examination should be performed followed by a colonoscopy in case of pathological findings in order to detect IBD-like disease in an early stage. This may allow appropriate treatment as soon as possible in the course of disease and prevent surgical treatment.

## Consent

Written informed consent was obtained from the patient for publication of this Case report and any accompanying images. A copy of the written consent is available for review by the Editor of this journal. The CARE guidelines have been used for this case report [17].

## Abbreviations

CT: Computer tomography
GSD: Glycogen storage disease
G-CSF: Granulocyte-colony stimulating factor
G(M)-CSF: Granulocyte-macrophage colony-stimulating factor
IBD: Inflammatory bowel disease
LLN: Lower limit of normal
TNF: Tumor necrosis factors
ULN: Upper limit of normal
